# An Intelligent Self-Service Vending System for Smart Retail

**DOI:** 10.3390/s21103560

**Published:** 2021-05-20

**Authors:** Kun Xia, Hongliang Fan, Jianguang Huang, Hanyu Wang, Junxue Ren, Qin Jian, Dafang Wei

**Affiliations:** Department of Electrical Engineering, University of Shanghai for Science and Technology, Shanghai 200093, China; xiakun@usst.edu.cn (K.X.); 182540397@st.usst.edu.cn (J.H.); 182540395@st.usst.edu.cn (H.W.); 182371464@st.usst.edu.cn (J.R.); 183780699@st.usst.edu.cn (Q.J.); 182540390@st.usst.edu.cn (D.W.)

**Keywords:** smart retail, self-service, internet of things (IoT), multi-device management, multi-object detection

## Abstract

The traditional weighing and selling process of non-barcode items requires manual service, which not only consumes manpower and material resources but is also more prone to errors or omissions of data. This paper proposes an intelligent self-service vending system embedded with a single camera to detect multiple products in real-time performance without any labels, and the system realizes the integration of weighing, identification, and online settlement in the process of non-barcode items. The system includes a self-service vending device and a multi-device data management platform. The flexible configuration of the structure gives the system the possibility of identifying fruits from multiple angles. The height of the system can be adjusted to provide self-service for people of different heights; then, deep learning skill is applied implementing product detection, and real-time multi-object detection technology is utilized in the image-based checkout system. In addition, on the multi-device data management platform, the information docking between embedded devices, WeChat applets, Alipay, and the database platform can be implemented. We conducted experiments to verify the accuracy of the measurement. The experimental results demonstrate that the correlation coefficient $R^{2}$ between the measured value of the weight and the actual value is 0.99, and the accuracy of non-barcode item prediction is 93.73%. In Yangpu District, Shanghai, a comprehensive application scenario experiment was also conducted, proving that our system can effectively deal with the challenges of various sales situations.

## 1. Introduction

As the most vigorous format in the retail industry, retail stores have been a hot topic under the sluggish traditional retail environment in the past two years. Retail settings are being challenged to become smarter and to provide greater value for both consumers and retailers [1]. The emergence of unmanned retail stores accelerates the checkout process and improves customer convenience. “Unmanned retail store” is a model that provides consumers with “self-service shopping”, and the user’s purchase process is not participated by any sales staff. For example, at the end of 2016, in Seattle, Amazon released Amazon Go’s smart concept store [2], and Alibaba Group opened its intelligent store “Taocafe” in Hangzhou city in July 2017 [3]. Customers only need to be authenticated through their smartphones at the entrance and then no explicit checkout procedures are needed before leaving. The “Just Walk Out” shopping concept was put forward, which means people can get goods quickly without queuing or checking out. To achieve “Just Walk Out”, advanced technologies are required to be integrated, such as computer vision, sensor fusion, and deep learning.

Automatic checkout (ACO) is one of the critical problems in the retail domain which aims to automatically generate the shopping list from the images of the purchase products. The target detection network can solve this problem. To achieve the goal of deploying to mobile devices, the object detection networks are expected to be not only accurate enough but also fast and small in size [4]. MobileNet-SSD [5], derived from VGG-SSD [6], has fewer parameters and better detection speed and is widely used in embedded systems. Paper [7] launched an accelerated checkout system, providing customers a fast and convenient checkout transaction by capturing barcodes and images through over a dozen cameras. It utilizes image recognition in an accelerated checkout system. Nevertheless, items have to be spaced separately onto the belt, simplifying the difficulty of identification. Moreover, the system requires too many cameras, making it expensive.

Currently, Radio Frequency Identification (RFID) technologies are employed to identify, capture, and transmit information [8]. Image-based action recognition is developed to detect taking and placing items from shelves into shopping carts [9]. Most of these services are combined with RFID sensors so that purchased items can be identified [10,11]. Paper [12] proposed a centralized and automated billing system using RFID and ZigBee communication. The authors of [12] used ZigBee technology to upload data periodically. For each product of shopping mall, supermarkets will be provided with an RFID tag, to identify its type, but adding tags and sensors is expensive. This method is not suitable for low-cost products. Paper [13] proposed a smart shopping cart equipped with a camera, the cart can monitor shopping activities and transfer the images to the cloud for processing, so that items in the cart can be tracked and checked out. It replaces RFID sensors with cameras. However, its application premise is that there is a one-to-one relationship between product and price, and it is not suitable for non-barcode products sold by weight.

Electronic balances have become so sophisticated that many calibrate themselves and appear to provide “error-free” measurements [14]. However, in the sales scenario, electronic balances require manual adjustment of product prices, and it is difficult to persistently store detailed transaction information. Internet of Things (IoT) and new ubiquitous connectivity paradigms beyond 5th-generation mobile networks (5G) have created unprecedented dynamics for opportunistic sensing by exploiting low-cost radio devices [15,16]. IoT technology allows different devices to access the internet, gives users feedback information, and receives and executes remote control commands from the client. Customer shopping information can be transmitted to the cloud database through wireless communication. The cloud server can effectively store and manage a large amount of device information [17]. Paper [18] proposed a wireless electronic weighing scale designed with an ultra-low-power MSP430 controller and ZigBee wireless communication module. Through ZigBee, consumer habits can be analyzed and inventory can be recorded in detail. This method is suitable for home and small test sites. Additionally, products are still marked manually.

For non-barcode goods, additional labor and material costs are consumed by weighing in advance and labeling with barcodes. The sales data are not connected to the Internet, and merchants are stuck in the timely revision of sales strategies. At the same time, the inability of real-time weighing limits the self-service cash register, which is not conducive to the development of smart retail. In this paper, an intelligent self-service vending system (ISSVS) is proposed to address the non-barcode products to the current image-based checkout system and sold them by weight. By building a convolutional neural network model on the Caffe-Jacinto platform, the system utilizes a lightweight convolutional neural network based on MobileNet to extract features from the sample data and uses an SSD target detector to identify the sample data. Online payment becomes possible by invoking Alipay face-to-face interface. Using weight sensors on each device, our system extracts the real-time mean and variance of the item being taken based on absolute weight change, which is fused with visual object identification once the item is on the pan. ISSVS leverages IoT to manage information, such as sales volume, turnover, so that the merchants can supervise the sales situation. To evaluate the system in a real-world setting, we conducted a trial experiment in retail stores in Yangpu District, Shanghai.

In summary, the contributions of this paper are as follows:

- We propose an intelligent self-service vending system that can detect multiple unlabeled products in real-time. The system realizes the integration of non-barcode items weighing, identification and settlement.
- We design a multi-device management platform through the IoT technology and achieved information docking between embedded devices, WeChat applets, Alipay, and database platforms.
- We propose a flexible weighing structure. The touch screen and camera are rotatable so that the system can recognize fruits from multiple angles and realize ACO for people of different heights.
- We propose to apply target detection in the sale of unlabeled products, and we manufacture and share 5637 tagged images of 16 unlabeled products.
- We carry out a comprehensive application scenario analysis that demonstrates that our system can effectively cope with the challenges of various sales situations.

The remainder of this paper is organized as follows. In Section 2, we discuss related works and background. In Section 3, we introduce the proposed system in detail from the aspects of hardware, software, and machinery. Section 4 gives a brief introduction to the application of our system in the IoT. The details of our experiments and results are presented in the Section 5. The last section concludes this paper with a discussion of the results.

## 2. Related Work

### 2.1. Image Processing

In recent years, deep learning has been successfully applied to image classification [19,20,21]. Deep learning can simplify or even omit the pre-processing of data, and directly use the original data for model training. It is composed of multi-layer neural networks to solve the defects in the traditional machine learning methods of artificial feature extraction and optimization, which extracts the features from the original data from low level to high level, from concrete to abstract [22].

Convolutional Neural Network [23] (CNN) is currently the most widely used multi-layer neural network in image recognition. CNN can directly take two-dimensional images as input, avoiding the complicated preprocessing process. The essence of CNN is to share parameters in multiple spatial locations, reducing the connection between each layer of the network and the number of training parameters, and improving generalization performance. In addition, the convolutional neural network has also obtained more significant performance than traditional mode methods in various applications such as face recognition [24], pedestrian detection [25], and speech recognition [26].

Traditional image recognition mainly includes four links: image acquisition, image preprocessing, feature extraction, and pattern matching, while the main complex work focuses on the image preprocessing and feature extraction stages. The authors of [27] proposed a detection method that combines Fast R-CNN with an adaptive threshold intuitionistic fuzzy set to segment and recognizes overlapping or occluded tomatoes on plants.

In the retail domain, some previous work has tackled the problem of item identification. The authors of [28] proposed to estimate object scales of images before generating object proposals, especially for supermarket images where object scales are usually within a small range. The authors of [29] developed a deep learning-based pipeline to solve the identification of grocery products on store shelves and achieve seamless processing of new products/packages.

In most of the recognition experiments, the image background is single, and the image data collection is completed in the same experimental environment, avoiding the changes in light intensity, shadow depth, and occlusion during collection, eliminating the influence of the external environment on the image, and the use of algorithms to delete complex image backgrounds improves the accuracy of image recognition.

### 2.2. Target Detection

The task of object detection is to find all the objects of interest in the image, determine their category and location, which is one of the core problems in the field of computer vision. Since various objects have different appearances, shapes, and postures, coupled with the interference of factors such as illumination and occlusion during imaging, target detection has always been the most challenging problem in the field of computer vision. Recently, based on the advancement of Graphics Processing Unit (GPU) technology, deep learning networks that extract features and detect objects have become the mainstream of image processing.

The three common target detection models for CNN are Faster R-CNN [30], You Only Look Once (YOLO) [31], and Single-Shot Detector (SSD) [6]. Faster R-CNN was proposed to achieve end-to-end detection for objects [30]. In Faster R-CNN, a Region Proposal Network (RPN) is used to produce high recall region proposals. The accuracy of models is higher than that of the traditional algorithms, but the detection speed is slow and cannot meet the real-time requirements. In 2016, YOLO [31] transforms the detection problem into a regression problem. The YOLO network draws on the GoogLeNet classification network structure. YOLO can detect objects very quickly, and the standard version of YOLO can reach 45 FPS on Titan X GPU [31]. However, the YOLO network does not use the region proposal, so the accuracy is not high when detecting small targets. In 2016, SSD [6] was proposed. SSD can be understood as the evolutionary method of YOLO and Faster R-CNN. SSD has the characteristics of multi-scale, which can examine the convolution results of different levels in the network at the same time and improve the detection speed.

## 3. System Architecture

To the best of our knowledge, ISSVS is a fully autonomous non-barcode items vending system without humans in the loop. This section provides our system design choices and assumptions.

### 3.1. System Overview

**Figure 1:**
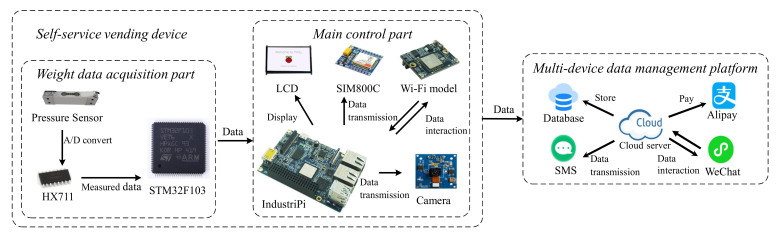
The architecture of the system.

The architecture of the proposed system framework is presented in Figure 1. ISSVS includes a self-service vending device and a multi-device data management platform. The self-service vending device is divided into a weight data acquisition part and the main control part; the weight data acquisition part obtains the weight of the goods so that the goods can be sold by weight; equipped with a mechanical casing to support image acquisition, the main control part employs deep learning technology to identify non-barcode items. Then, the information interaction between the main control part and the weight data acquisition part could be accomplished by the serial communication port. The main control part utilizes the Transmission Control Protocol (TCP) to periodically transmit order operation parameters to the cloud server via the Wi-Fi module. In addition, the multi-device data management platform interacts with the WeChat applet and Alipay.

### 3.2. Main Control Part

The main control part has modules such as an IndustriPi (Jiang-niu, Beijing, China), a camera, a Wi-Fi module, an SIM800, an LCD module, and so on. As the core of the entire system control, the IndustriPi realizes the multi-object detection, weight data interactive function, the cash register function, and the cloud interaction function. IndustriPi is an open-source intelligent hardware development platform based on Texas Instruments’s AM5708 (Texas Instruments (TI), Dallas, TX, United States), which is a heterogeneous multi-core processor. AM5708 is based on the ARM Cortex-A15 core with a dominant frequency of 1 GHz. The memory is shared between ARM core and DSP core, which realizes the low-latency characteristics of chip processing. IndustriPi can be applied in various industrial monitoring applications to meet the actual needs.

The main control part utilizes modular design ideas to compile different functions to a clear data flow. The program could be divided into UI interface display, interactive subroutine with the weight data acquisition part, interactive subroutine with cloud server, and image processing subroutine. Data sampling is set to the highest priority to collect weight data in real-time.

**Figure 2:**
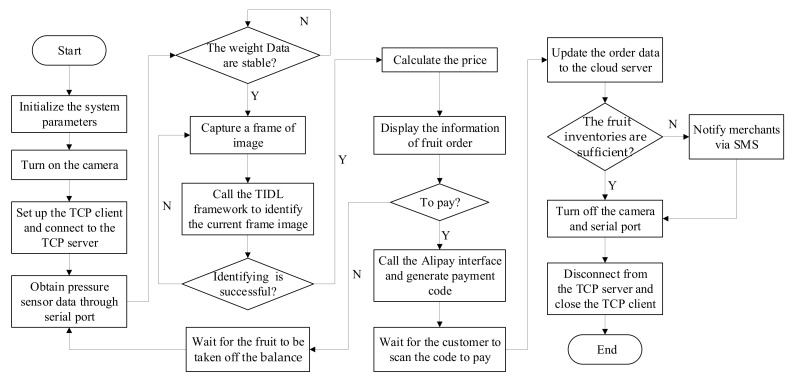
The flow chart of the main control part.

The flow chart of the control part is shown in Figure 2. ISSVS’s camera will be triggered when the change of the total weight of the load cell is detected to be greater than a certain threshold, and then IndustriPi will recognize the current image automatically by calling the deep learning model. The recognition results are judged according to the sales principle, that is, only when the identification results belong to the same category can the purchase operation be carried out; otherwise, it will prompt “Please make sure to place the same product on the pan”. After successful identification, the total price of non-barcode items will be calculated according to the weight and unit price information; the order is generated and displayed on the touch screen. Users could verify the shopping cart to confirm the recognition results. If there is a recognition error, the error result can be deleted from the shopping cart and the purchased items can be reidentified. When the settlement button is pressed, a Quick Response code (QR code) will be popped up, and users could complete payment for commodities through Alipay. The order will be synchronized to the cloud database for storage and update. A successful payment window will be popped up on the screen, and the initial interface will be loaded to wait for the next weighing and payment. In addition, with the help of the SIM800 module, the system notifies the merchant to replenish the goods via SMS.

### 3.3. Weight Data Acquisition Part

**Figure 3:**
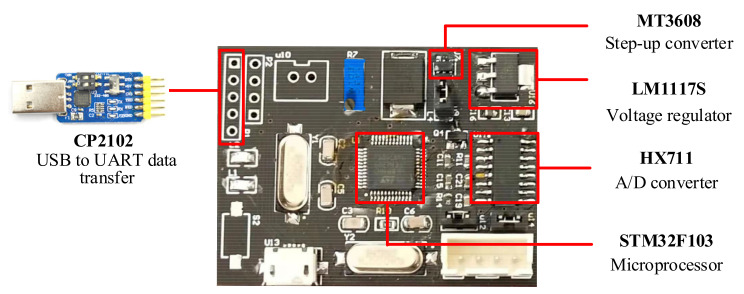
Circuit board integrated with the weight data acquisition part. The voltage level of the hardware platform is 5 V and the regulator chooses the low dropout regulator (LDO) LM1117. USB to UART data transfer is a modular structure, inserted into the circuit board.

A new integrated weight data acquisition platform has been designed to achieve a miniaturized system (Figure 3). The weight data acquisition part is composed of a microprocessor, an analog/digital (A/D) conversion module, and a load cell.

As the microprocessor of the weight data acquisition part, the STM32F103 series chip in the 48 PIN package is applied, which is based on the medium-performance ARM Cortex-M3 32-bit RISC core with a dominant frequency of 72 MHz. The microprocessor offers numerous peripherals, standard and advanced communication interfaces [32], such as three 12-bit analog-digital converters (ADC), two 12-bit digital-analog converters (DAC), and 15 general-purpose timers, communication interfaces (USART, I2C, SPI, and Ethernet).

The weak voltage signal can be transformed from analog signal to digital signal by A/D converter chip HX711. The chip, designed for a high-precision electronic scale, integrates peripheral circuits such as a regulated power supply circuit, which has the advantages of high integration, fast corresponding speed, and strong anti-interference ability. Within the measurement range of 10 kg, the measurement accuracy of HX711 with 24-bit resolution reaches milligram level.

The resistance strain gauge load cell is used to obtain the weight of non-barcode items. The range of the load cell is 0~10 kg. The sensitivity is 1.0 ± 0.1 mV/V, and the composite error is 0.05% FS. The parameter index of the load cell is shown in Table 1.

**Figure 4:**
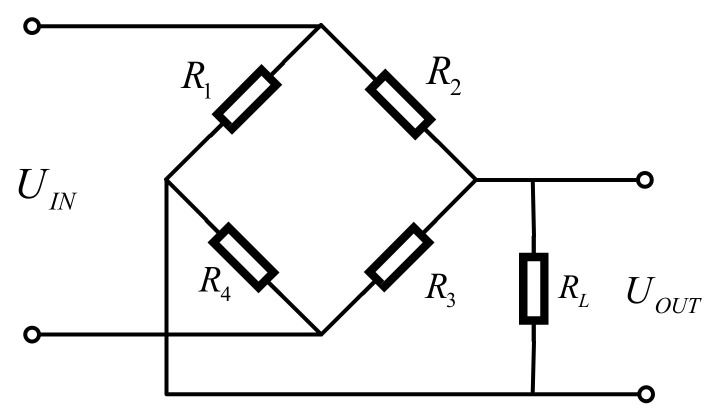
Circuit schematic diagram of Wheatstone bridge.

Wheatstone circuit is used to convert the signal of the resistance strain gauge load cell, and the circuit diagram is shown in Figure 4. The sampling circuit is employed to collect the signal of the Wheatstone bridge and the differential amplifier circuit is adopted to suppress the common-mode interference. When in the same temperature field, the effects of temperature are canceled out and the voltage output sensitivity is high [33].

The measurement circuit has four bridge arms consisting of $R_{1}$,$\;R_{2}$,$\;R_{3}$, and $R_{4}$. The output voltage is shown in Formula (1):

(1) $$U_{OUT}=\left(\frac{R_{1}}{R_{1}+R_{2}}-\frac{R_{4}}{R_{3}+R_{4}}\right)U_{IN}$$

where $U_{IN}$ is the excitation voltage.

When all the strain gauge resistances are R, the bridge is an equal-arm bridge. $R_{1}$,$\;R_{2}$,$\;R_{3}$, and $R_{4}$ are regarded as $R+\Delta R_{1}$, $R+\Delta R_{2},$ $R+\Delta R_{3}$, and $R+\Delta R_{4}$. $U_{OUT}\;$ is shown in Formula (2):

(2) $$U_{OUT}=\left(\frac{R+\Delta R_{1}}{2R+\Delta R_{1}+\Delta R_{2}}-\frac{R+\Delta R_{4}}{2R+\Delta R_{3}+\Delta R_{4}}\right)U_{IN}$$

The weighing errors of load cell include zero drift error and repeatability error, etc. The load cell is affected by different sizes and multiple reciprocating impact loads during operation. Due to the non-standard force, eventually the contact of the sensor changes. The zero deviation of the sensor and amplifier will cause zero deviation of the measurement result. To avoid zero drift error, we construct a weight calibration technology. When the weight data acquisition starts, we ensure no load is placed on the pan. The weight data acquisition part collects the initial weight value in real-time and takes the average of 20 parameters within 2 s as the reference value at zero. Under the same load and the same environmental conditions, the repeatability error is the difference between the outputs of several successive experiments. Repeatability error is caused by some relatively fixed factors, such as changes in environmental conditions such as temperature, humidity, wind, and gravity field during weighing measurement. To reduce repeatability error, a sliding window filter with a width of 24 is applied to the filter parameters. The real-time mean and variance are calculated to suppress the small-amplitude noise and improve the robustness of the system.

### 3.4. Vision-Based Object Identification

#### 3.4.1. Data Collection

For achieving good recognition accuracy, huge datasets are required. We construct a market database as the training set to facilitate the research of product recognition with emerging deep learning techniques. We utilize the 8 million pixels camera as an image capture device for 16 kinds of non-barcode items. These images require considerable images of each object, under different conditions, with different backgrounds, and from different angles. Due to the particularity of the usage scenario, the data acquisition is manipulated in the environment of outdoor natural illuminant in the daytime and indoor powerful illuminant in the night. Optimal lighting might help get sharper and more consistent views of the products.

**Figure 5:**
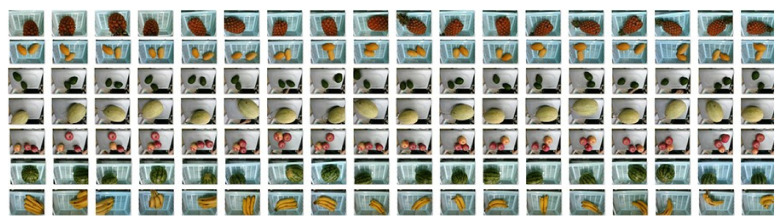
The sample data of the database. The database of this paper could be obtained through the website: https://github.com/ClaireFan421/dataset-fruit029.git (accessed on 29 October 2020).

Data augmentation is common in data collection. It not only increases noise data but also improves the generalization ability and robustness of the model. The specific performance of data augmentation is as follows: data rotation, horizontal migration, vertical migration, etc. [22]. A total of 5637 images of non-barcode items are collected, of which 3253 are divided into the training sets and the rest are divided into the testing sets. What is more, to speed up the processing of images, these images are resized into 300 (pixels) × 300 (pixels) in the experiment. The sample data of the database are presented in Figure 5.

#### 3.4.2. Data Labeling

**Figure 6:**
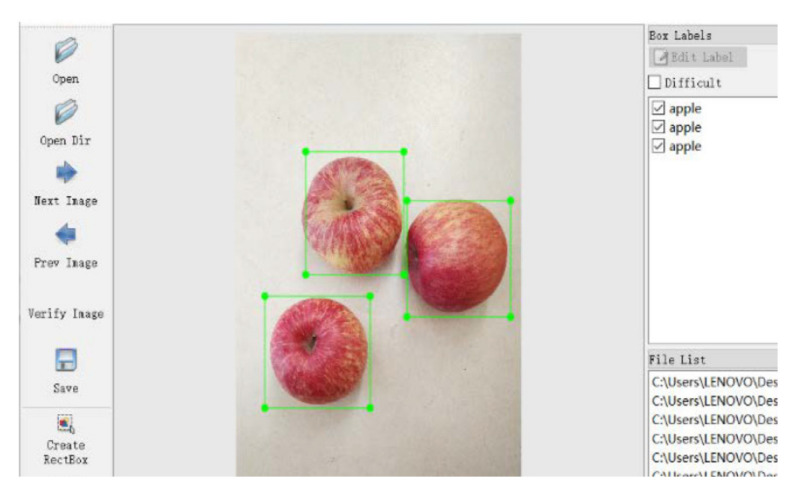
The annotation of the dataset. The ground truth of apple is green bounding boxes. As an image annotation tool, LabelImg can save the generated annotation as an XML file in Pascal VOC format without secondary conversion.

These images require labeling of the ground truth for training; this could be done manually, creating a labor-intensive problem [34]. The bounding box is one of the most common types of image annotation in computer vision. It refers to drawing a rectangular box around the detected object. In this paper, the boundary box is applied to label the image, and LabelImg is chosen for image annotation. The annotation of the dataset is presented in Figure 6.

#### 3.4.3. Object Detection

Responding to the need for real-time applications and fast recognition, a lightweight model with low delay is expected. The SSD model structure includes VGG convolutional structure, convolutional layer, and multi-scale classifier, among which VGG convolutional structure is used for feature extraction [35].

**Figure 7:**
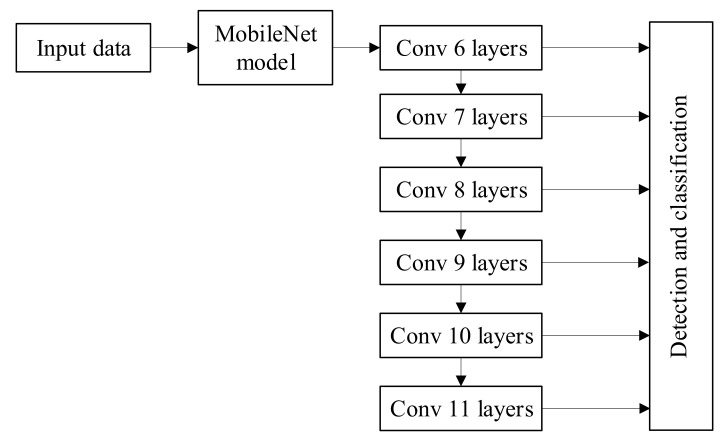
The MobileNet-SSD object detection model.

MobileNets is an efficient CNN designed for mobile and embedded vision applications [36]. MobileNets uses depth-wise separable convolutions to decompose the standard convolution filter into a depth convolution filter and a 1 × 1 convolution filter, and constructs lightweight deep neural networks based on a streamlined architecture [36]. The MobileNets can effectively reduce the computational complexity of convolution operations and the complexity of neural network models. The lightweight model MobileNets is chosen as the feature detector of the SSD to achieve multi-stage object detection [36]. The last average pooling layer and full connection layer of MobileNets are converted into a multi-scale feature mapping layer of the SSD algorithm [5]. MobileNet-SSD only uses convolution filters of 3 × 3 and 1 × 1 sizes, which reduces the cost of computation while ensuring a similar detection effect. It solves the problem that SSD cannot run on mobile devices [31]; it was unparalleled in terms of detection speed due to the substantial reduction of its parameters. The MobileNet-SSD object detection model is shown in Figure 7.

Network objective loss function includes positioning loss function and regression loss function [37]. These two functions determine the accuracy of the network structure during the training process, and the loss function updates the network parameters [31]. The loss function is shown in Formula (3):

(3) $$L\left(x,c,a,b\right)=\frac{1}{N}\left(L_{conf}\left(x,c\right)+\gamma L_{loc}\left(x,a,b\right)\right)$$

where  N represents the number of unit boxes; the initial value of the weight γ is 1; x is the center coordinate, a is the width value, b is the length value, and c is the confidence level.

**Figure 8:**
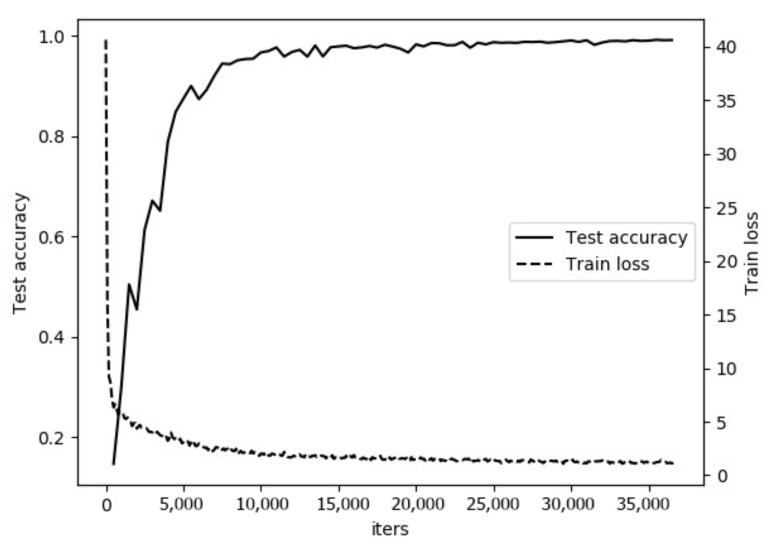
The loss and accuracy curves of models as the number of iterations increases.

Figure 8 shows the loss and accuracy of models as the number of iterations increases. The loss tends to stabilize at about 15,000 epochs and the value of classification accuracy is finally stable at about 98%. After testing, it takes 30.7228 ms to do a forward inference time per image. The above verifies that the model can be successfully applied in non-barcode item recognition problems.

#### 3.4.4. Texas Instruments Deep Learning

**Figure 9:**
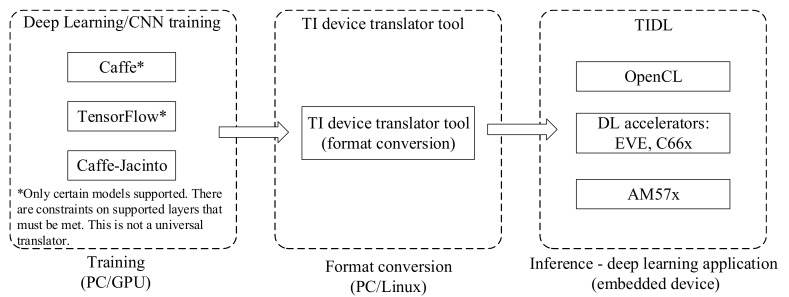
TI Deep Learning (TIDL) development flow. Deep learning consists of two stages: training at the development stage and inference at the deployment stage. Training involves designing the neural network model, running training data through the network to tune the model parameters. Inference takes the pretrained model including parameters, applies to new input, and produces output. Once the network is trained, the TIDL converter tool can be used to translate the network and parameters to TIDL.

The IndustriPi supports Texas Instruments Deep Learning architecture (TIDL). TIDL is a set of open-source Linux software packages and tools, based processor SDK Linux 5.0, and TIDL brings deep learning to the edge by enabling applications to leverage TI’s proprietary, highly optimized CNN implementation on the EVE and C66x DSP compute engines [38]. The MobileNet-SSD model, optimized by TIDL, is run in the DSP calculation engine in the IndustriPi to realize the recognition of non-barcode items [39]. Figure 9 shows the TIDL overall development process.

### 3.5. Mechanical Structure

**Figure 10:**
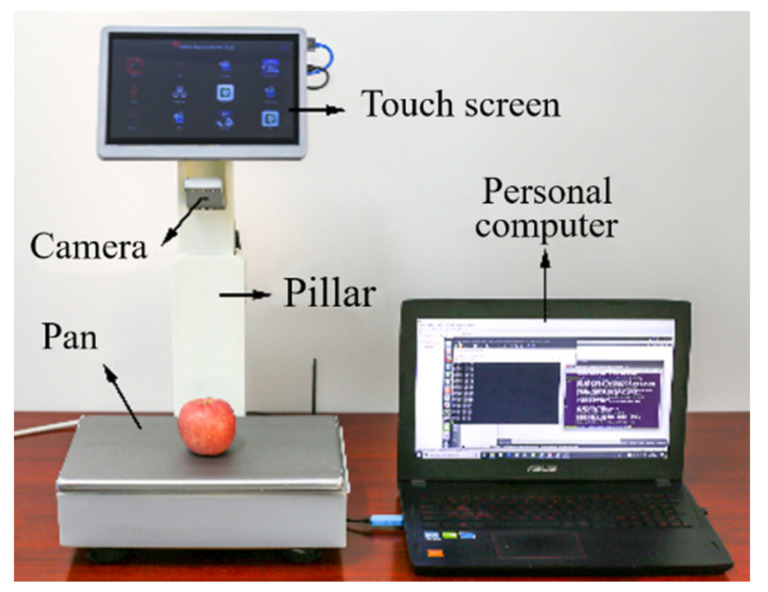
The entire mechanical structure of the ISSVS prototype.

The mechanical structure is mainly composed of a display screen bracket, a camera, a pillar, and a base structure, as shown in Figure 10. The connection part of the touch screen bracket and the pillar is an asymmetric hollow structure that reduces the overall weight of the system. The touch screen and the camera are connected to the pillar through a damping shaft, and the camera can be rotated to recognize non-barcode items from different angles. The pillar with a slide rail is telescoped to adjust the height of the camera and touch screen. SolidWorks software is employed for the computer-aided design in this study, and we establish a three-dimensional (3D) model. At the same time, industrial-grade SLA 3D printing technology is utilized to process the mechanical structure. The mechanical specifications of ISSVS are shown in Table 2.

## 4. IoT Applications

**Figure 11:**
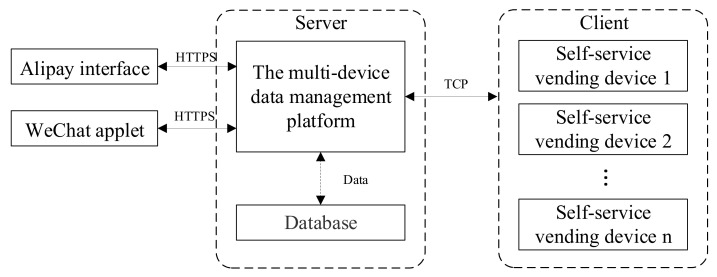
The flow of information inside the IoT platform.

The IoT terminal based on the Alibaba Cloud IoT platform can be divided into a 4-layer architecture: IoT platform, application layer, edge node, and edge device [40]. The IoT platform provides a safe and reliable connection and communication with the device. In addition, the IoT platform can receive data from devices and distribute data to devices. In the application layer, applying the services provided by the IoT platform, the running status of the platform is exhibited in the visual interface, and the management instructions are transmitted. Edge node refers to the business platform constructed on the edge of the network close to the user, providing storage, computing, network, and so on. The edge nodes process and upload data of edge devices. Edge devices, considered to have no computing power, are data producers in the IoT system. The flow of information inside the IoT platform is shown in Figure 11.

As a mobile device, WeChat mini program accesses a multi-device data management platform through Hypertext Transfer Protocol on a secure socket layer (HTTPS) for data monitoring and management. The Alipay interface is invoked to implement online payment. Through TCP/IP, the multi-device data management platform is accessed by clients. In this paper, the self-service vending device could be regarded as the edge node, the application layer is composed of a WeChat applet and an Alipay interface.

### 4.1. Multi-Device Data Management Platform

**Figure 12:**
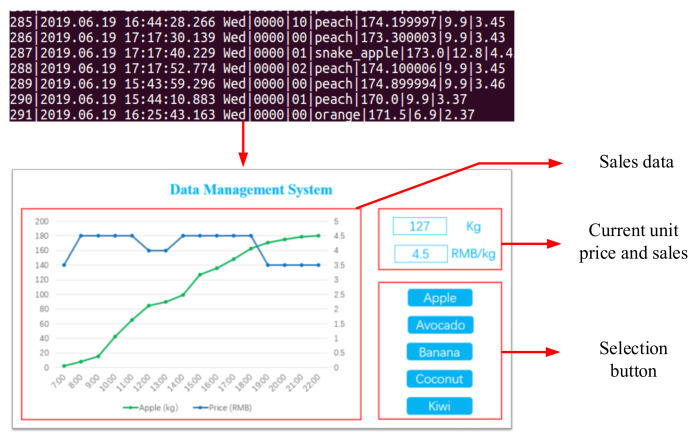
The terminal interface of the multi-device data management platform. By calling the cloud database data, the daily sales curve of different products can be visualized in the multi-device management platform, such as the inventory and sales price.

The multi-device data management platform, designed for merchants, employs the Alibaba Cloud IoT platform to mine data. The core of the multi-device data management platform is the web visual interface, which can remotely monitor the real-time measurement data of multiple devices and output historical sales data. The terminal interface of the multi-device data management system is shown in Figure 12.

The database is created in the server, and a PHP script is used to open the HTTPS access interface for the database [41]. Devices and users access the database through the HTTP protocol. Users need to select the device to be viewed through the user interface and then they can view the unit price fluctuation and real-time sales data of the selected device. Historical sales data can be displayed in the form of data tables or line charts.

### 4.2. Payment Interface

**Figure 13:**
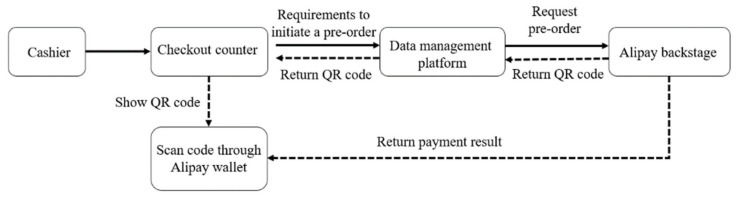
The flow chart of the electronic payment process.

By invoking the face-to-face interface of Alipay, online payment is applicable to the ISSVS. Alipay has become a powerful mobile payment platform in the market with more than 1 billion users [42]. There are two operation methods for online payment: “active scan” and “passive scan”. The fundamental difference between them lies in the information contained in the QR code. “Active scan” means that consumers apply their mobile phones to scan QR codes that provide payment information. “Passive scan” refers to a QR code supplied by a customer, the merchant applies a scanner to scan it. We utilize the active scan mode to complete the payment process. The flow chart of the electronic payment process is shown in Figure 13.

**Figure 14:**
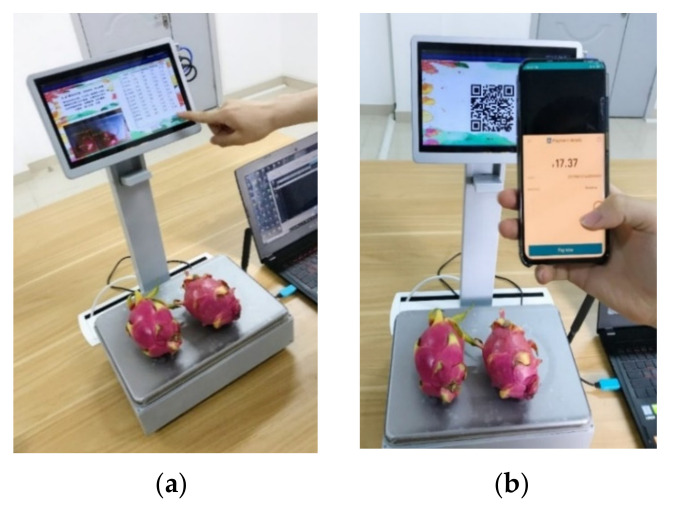
The payment process of the intelligent self-service vending system. (**a**) Click the checkout button; (**b**) pay with Alipay application.

The IndustriPi invokes Alipay’s face-to-face payment application programming interface (API) through HTTPS. The self-service vending device creates order information and transmits the request to Alipay. The payment QR code is generated according to the payment Uniform Resource Locator (URL) returned by Alipay. Customers can scan the QR code to transact. After payment, Alipay will transmit a message to the platform to notify the payment of success or failure. If the payment is successful, the home page will be loaded on the interface, and the self-service vending device will wait for the next settlement; otherwise, the interface will be in payment status. The actual payment steps are shown in Figure 14.

### 4.3. WeChat Mini Program

**Figure 15:**
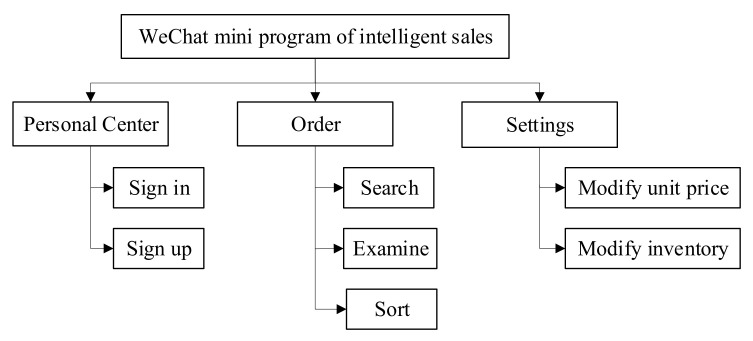
The functional block diagram of the mini program.

Tencent’s fourth-quarter financial report shows that in 2020, WeChat, as an instant messaging software, has more than 1225 million monthly active users, ranking third in the world, only behind WhatsApp and Messenger [43,44]. Unlike applications that need to be compatible with Android and IOS systems, mini-programs rely on WeChat, which is a program that does not need to be downloaded. WeChat mini-program, closely combined with intelligent sales business, realizes data visibility, commodity management, and market analysis on the mobile terminal. Hypertext preprocessor (PHP) script language is applied in the development of the WeChat applet and PHP code is invoked in static web pages embedded in HyperText Markup Language (HTML). The development environment of PHP 5.6, SQLite3, and Apache are constructed in the integration software package XAMPP to optimize the running environment. The functional block diagram of the mini-program is shown in Figure 15.

**Figure 16:**
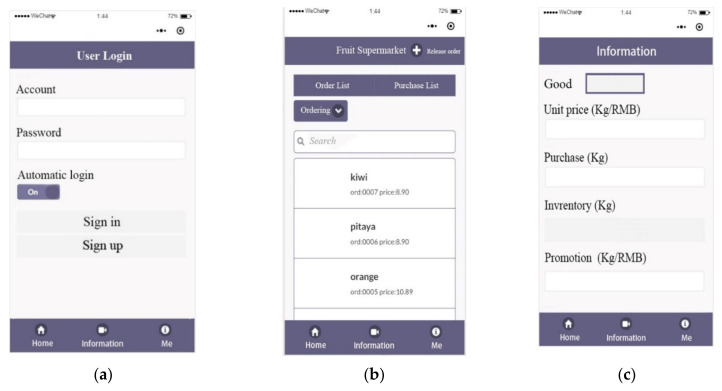
The design interfaces of mini-program. (**a**) Login interface. The username and password interact with the server by submitting a form. Only after the server-side verification is successful can the order information page of the applet be accessed; (**b**) order interface; (**c**) setting interface.

The interfaces of the mini-program are composed of personal center, order, and settings. Only after login and authentication can the order information and inventory information be visible. The order interface provides the search function. Users can load the order to facilitate the inventory verification by searching the order number or order time. The market could be analyzed by the visualization of sales volume and turnover. In the setting interface, the merchant can manage the goods by modifying the unit price and inventory of the goods. The sales strategy can be revised in real-time to obtain optimum sales, according to the information of customer demand and purchasing power in different periods. The design interfaces of the mini-program are shown in Figure 16.

## 5. Experiments and Results

The experimental session evaluates the measurement and verifies the accuracy of target detection in a real environment; at the same time, the application of the system in the actual sales environment is verified.

### 5.1. Measurement Experiments

There are three crucial parameters of electronic balances: readability, repeatability, and linearity. The readability is the scale division value of the electronic balances, and the division value is related to the maximum range and the sensor resolution. When a 24-bit load cell is selected, the division value can be calculated as Formula (4):

(4) $$d=\frac{Y}{2^{24}}$$

where Y is full-scale output. This way, when the full scale is 10 kg, the division value is 0.6 mg.

Repeatability consists of loading the balance with the same load under repeatability conditions that include the same measurement procedure, same operators, same measuring system, same operating conditions, and same location and replicate measurements on the same or similar objects over a short period [45].

Under specified conditions, linearity is the ratio of the maximum deviation between the calibration curve and the fitted curve of the sensor to the full-scale output. Linearity is also known as “non-linear error”; the smaller the linearity value, the better the linearity characteristic. Linearity can be calculated as Formula (5):

(5) $$\delta =\frac{△Y_{max}}{Y}\times 100\%$$

where △$Y_{max}$ is the maximum deviation.

**Figure 17:**
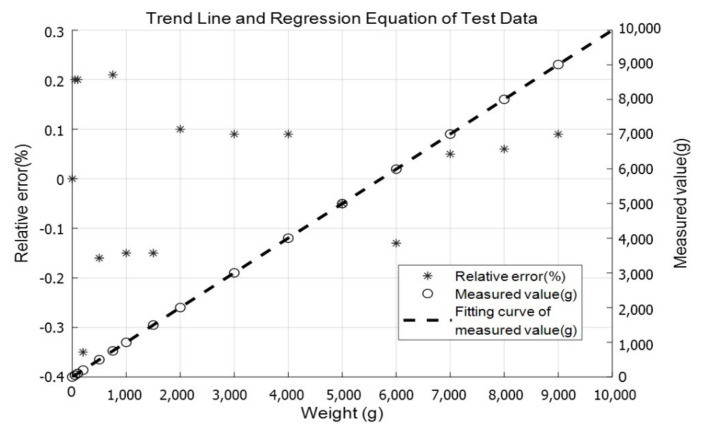
Trend graph of the measured value and relative error.

After turning on the power of the load cell, the weight data acquisition part first performed the zero adjustment and then placed the standard weight in the center of the tray. When the data were stable, the displayed value was written down, and then the mass of the weight was increased in turn. The test weight should be measured multiple times in the same order, that is, ascending or descending weight order of weight. We performed the same load multiple repeated measurements, and calculated the average value of the difference as the repeatability parameter value. The test results were processed by MATLAB 2019 software, and linear regression analysis was performed. The measured values and error rate of the system are shown in Figure 17.

It can be seen that the relative error of the system within the range of 0–10 kg is not more than 0.4% FS, and the coefficient of determination for linear regression $R^{2}$ is 0.99. The results demonstrate that the weight data obtained by the ISSVS have fine linearity and measurement accuracy. The specifications of the ISSVS are shown in Table 3.

### 5.2. Image Recognition Experiments

In this part, we first describe the implementation details and parameter settings of the proposed method. Then, the identification accuracy verification and running state verification of the system are carried out. The operating system and deep learning platform used are Ubuntu 16.04 and Caffe-Jacinto, respectively. The model is trained on a computer with one NVIDIA GeForce GTX 1060 GPUs.

#### 5.2.1. Implementation Details and Parameter Settings

The experimental environment is composed of a single camera with a height of 24 cm and a depression angle of 26 degrees.

The parameter of batch size is set at 16 to ensure the highest recognition accuracy. For MobileNet, the initial learning rate of 0.0005 is lower so as not to disrupt the ImageNet weights, and it is dropped by 0.5 every 15,000 iterations. Root Mean Square Prop is selected as the optimization algorithm; it optimizes the problem that the loss function swings too much in the update, and further accelerates the convergence speed of the function. Finally, the Mobilenet-SSD network with 35,000 iterations is selected as the default configuration of the training network.

#### 5.2.2. Accuracy Verification of Single Variety Products

**Figure 18:**
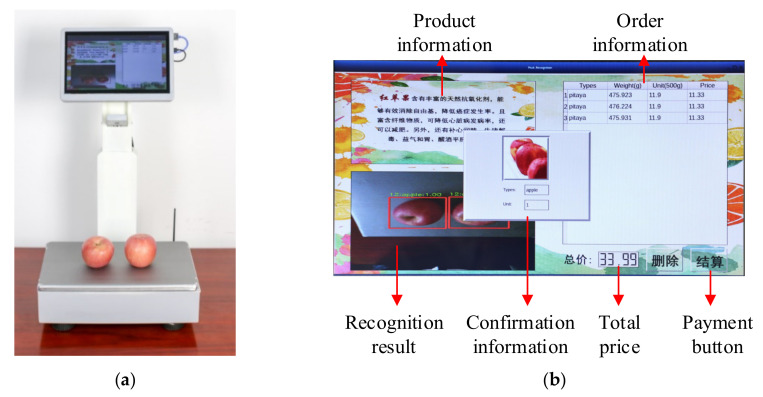
Identification test of single variety products. (**a**) Experimental environment; (**b**) the user interface after successful identification.

In the image recognition test, 16 sample data subsets are collected approximately, and each comprises 300 non-barcode test samples. It ensures that each sample contains the same item. The identification test of single variety products is shown in Figure 18. The product detection accuracy is shown in Table 4. According to the test in the verification set, the overall accuracy can achieve more than 93.73%, which verifies that the platform is accurate in identifying different kinds of non-barcode items.

#### 5.2.3. Running State Verification of Multi-Variety Product

**Figure 19:**
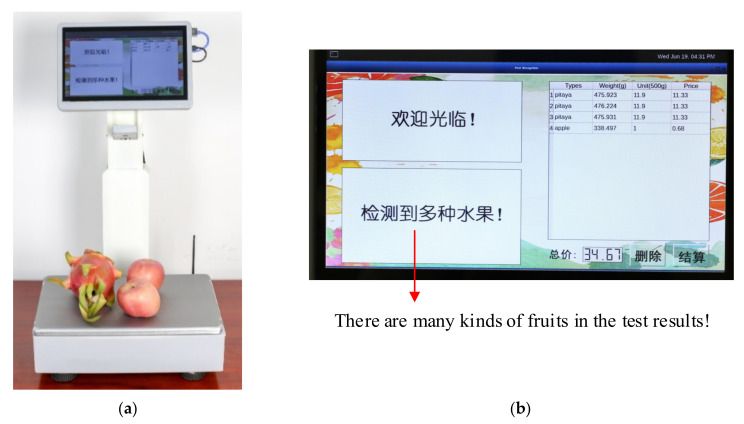
The identification test of the multi-variety product. (**a**) Experimental environment; (**b**) the user interface of multi-variety product testing.

According to the sales principle, a variety of items have their unit price, so they cannot be weighed at the same time. The multi-variety products were placed in the pan to check whether the system can detect any abnormalities. The identification test of multi-variety products is shown in Figure 19.

### 5.3. Real-Sales Experiments

To fully evaluate the system in a real-world setting, we conducted the system at a retail store in Yangpu District, Shanghai, and presented the results here.

#### 5.3.1. Assumptions

In order to ensure that the prototype can be experimented with in the real sales environment, we made the following assumptions:

- Model training is performed on the products that need to be detected to extract features of the products;
- The total weight of a single test product is within the range of the load cell;
- Alipay’s merchant account is an experimental account.

#### 5.3.2. Experiment Settings

The experiment we designed tried to simulate a real shopping experience, in which customers randomly choose what they want to purchase but may not know how to use the ISSVS. We experimented with eight participants between the ages of 20 and 40. At the beginning of each trial, the participants could select no less than five items (repetitions allowed). The participants verified the shopping cart to confirm the recognition results. After the trial, the participants paid for the purchased items. To compare with the manual settlement, with the help of the cashier, participants completed the payment with the same items.

#### 5.3.3. Experiment Results

In a large-scale traditional sales model, at least two staff members are required to be responsible for the sale. Staff 1 will label the unmarked items, and Staff 2 will price the labeled products at the outlet of the retail store (on small occasions, Staff 1 can complete the work of labeling and pricing at the same time). We record the time of single item identification and weighing and compare the time of manual weighing, pricing, and payment on small occasions. The experimental results are shown in Table 5. The weighing and recognition time for ISSVS is about 2 s, and the time for the system to wait for Alipay to callback payment information is about 1 s.

The experimental results show that consumers who have not used the system need to spend some of the time adapting to the system, which is determined by people’s acceptance of a new technology. Once participants use the system many times, the time of self-service service may be the same as that of manual service, even faster.

## 6. Conclusions

This paper introduces the intelligent self-service vending system which combines deep learning with retail. Sixteen kinds of non-barcode items can be identified by the trained MobileNet-SSD network. With the help of the Wi-Fi module, the sales data can be uploaded to the cloud database for storage and management. Merchants could acquaint the transaction, and develop a distinct sales strategy. By connecting Alipay’s secure and fast electronic payment interface, real-time and convenient payment is achieved. Through the multi-device data management platform based on the Alibaba Cloud IoT platform, multiple devices in supermarkets can be managed and monitored. The implemented prototype demonstrates that an efficient deep learning method, cloud computing, and fast network system are the key elements in developing a successful smart shopping platform. The intelligent self-service vending system based on artificial intelligence provides a retail solution for supermarkets without a human in the loop.

In future work, to identify more products, it is necessary to perform migration learning on the original recognition network so that the recognition network can capture the characteristics of new items. We will extend the categories of datasets and non-barcode items in the application scenario. A feedback application will be designed. The system will be able to fine-tune and update the training network according to the pictures provided by users in the feedback application so that the accuracy and generalization of the network will be improved.

## 7. Patents

The fresh food self-service selling device can be searched by publication (announcement) number CN210244529U.

## Figures and Tables

**Table 1:** Parameter index of the resistance strain gauge load cell.

Parameter	Value	Parameter	Value
Composite Error (%FS)	0.05	Input Resistance (Ω)	1000 ± 50
FSO (mV/V)	1.0 ± 0.1	Output Resistance (Ω)	1000 ± 50
Non-Linearity (%FS)	0.05	Insulation Resistance (Ω)	≥2000 (100 VDC)
Repeatability (%FS)	0.05	Operating Temp Range (°C)	−10~+50
Hysteresis (%FS)	0.05	Safe Overload (%FS)	150
Creep (%FS/3 min)	0.05	Excitation (V)	3~12 DC
Zero Balance (%FS)	±0.1	Maximum excitation (V)	15

FS represents the Full Scale.

**Table 2:** Mechanical Specifications of the ISSVS.

Range of Height	Maximum Width	Size of Screen	Screen Resolution	Size of Pan
44–54 mm	310 mm	250 × 170 mm	1024 × 600 pixels	340 × 234 mm

**Table 3:** Measurement specifications of the ISSVS.

Parameter	Value
Weighing Capacity (kg)	10
Readability (mg)	1
Repeatability (≤mg)	±2
Linearity (≤mg)	±0.1
Pan Size (mm)	280 × 200

**Table 4:** The accuracy of product detection.

Name	Accuracy (%)	Name	Accuracy (%)
Watermelon	89.52	Pitaya	91.23
Muskmelon	87.41	Hami melon	95.91
Coconut	92.92	Apple	89.36
Pineapple	95.87	Orange	96.19
Mango	95.78	Lemon	96.27
Kiwi	93.35	Carrot	96.34
Avocado	91.74	Potato	95.12
Banana	96.35	Tomato	96.38
Average Accuracy	93.73		

**Table 5:** The time comparison of different sales methods.

Category	Self-service Vending System	Manual Service System
Average Weighing and Recognition Time (s)	3	2
Average Alipay Callback Time (s)	1	1

## Data Availability

Not applicable.
